# Applying pH Modulation to Improve the Thermal Stability of Melamine–Formaldehyde Microcapsules Containing Butyl Stearate as a Phase-Change Material

**DOI:** 10.3390/polym16172463

**Published:** 2024-08-29

**Authors:** Branko Alič, Urška Šebenik, Matjaž Krajnc

**Affiliations:** University of Ljubljana, Faculty of Chemistry and Chemical Technology, Večna pot 113, 1000 Ljubljana, Slovenia; branko.alic@fkkt.uni-lj.si (B.A.); urska.sebenik@fkkt.uni-lj.si (U.Š.)

**Keywords:** phase-change materials, microencapsulation, etherified melamine–formaldehyde resin, thermal stability

## Abstract

This paper presents a two-stage microencapsulation process that uses pH modulation to enhance the thermal stability of microcapsules that consist of a melamine–formaldehyde (MF) shell and a butyl stearate core. In the first stage, the pH value was modulated between 6.0 and 8.0. Rising the pH value to 8.0 slowed the polycondensation rate, allowing the MF resin with a lower degree of polymerization to migrate to the capsule surface and form a smooth shell. Lowering the pH value to 6.0 accelerated polycondensation. In the second stage, a relatively fast, continuous reduction in the pH value to 5.0 led to further MF polycondensation, hardening the shell. Post-curing at 100 °C prevented shell damage caused by the liquid–gas phase transition of the core material during the process. The microcapsules produced by increasing the pH value to 8.0 twice demonstrated improved thermal stability, with only a minimal overall weight loss of 5% at 300 °C. Significant weight loss was observed between 350 and 400 °C, temperatures at which the methylene bridges in the MF shell undergo thermal degradation. The results from differential scanning calorimetry, electron microscopy, and thermogravimetry analyses confirmed a successful optimization of the microencapsulation, showing that these microcapsules are promising for thermal energy storage and other applications that require high thermal stability.

## 1. Introduction

Phase-change materials (PCMs) play a crucial role in regulating temperature and minimizing energy consumption in a variety of applications [1,2,3,4,5,6,7,8,9]. Their ability to absorb significant thermal energy during melting and release it during crystallization is essential for their functionality. However, suitable immobilization is required to prevent leakage due to their transition from solid to liquid. Leakage or diffusion of PCMs can severely impair their heat storage efficiency and cause risks such as fire hazards [10] and environmental pollution.

Microencapsulation is one of the several methods available for preventing this kind of leakage and offers additional benefits. Not only does it provide a well-defined, large surface area that enhances the heat exchange efficiency, but it also allows the PCM to be dispersed as a filler material in wall-building materials such as concrete, plaster, various foams, and even thin films like coatings [11,12,13,14,15]. Moreover, the stiffness and thickness of the microcapsule shells can be optimized for specific applications by adjusting the materials and process parameters used in the microencapsulation [16,17,18,19,20].

Butyl stearate [21,22,23,24,25] is one of the sustainable, bio-based PCMs [26,27] suitable for use in thermal energy storage systems operating at ambient temperatures. Its suitability is due to its melting/crystallization temperature, high melting/crystallization enthalpy, and production from renewable resources. Melamine–formaldehyde (MF) resin is commonly used as a shell material for PCM microcapsules. Cured MF resin exhibits exceptional stiffness, hardness, thermal stability, and chemical resistance, while also acting as a flame retardant [28,29,30]. Several research groups have reported success in producing mechanically stable MF microcapsules with excellent thermal stability. These results have been achieved through various methods, including continuous or stepwise additions [31,32,33] of MF resin to the reaction mixture, modification of the MF resin [34], the addition of co-reactants [18,35,36], and other shell modifications [37,38,39,40,41,42,43,44,45].

MF microcapsules can be prepared through the in situ polycondensation of MF prepolymers or their derivatives in an acidic environment. Under these conditions, the protonation of hydroxyl groups enhances their ability to act as leaving groups, thereby facilitating the condensation process. Achieving the desired properties of the microcapsules requires careful selection of the process parameters during microencapsulation [30,46,47,48,49,50,51,52,53,54] and precise adjustment of the MF prepolymer’s properties, including its chemical composition, reactivity, and degree of polymerization [55,56,57,58]. These properties can be tailored during the synthesis of the MF prepolymer, which is usually carried out in an alkaline medium. In the first step, formaldehyde and melamine react to form methylolmelamines, followed by condensation reactions in the second step [59].

The extensive condensation of the MF prepolymer occurs during microencapsulation. As the methylol groups react with each other to form methylene ether and methylene bridges, the degree of polymerization increases. Further reactions of these longer chains lead to crosslinking, resulting in a highly crosslinked three-dimensional network structure. This highly crosslinked network imparts excellent heat and mechanical resistance to the resin.

As the degree of polymerization of the MF prepolymer increases, its solubility in water decreases, leading to the collapse of longer polymer chains onto the surface of the hydrophobic PCM droplets. Initially, the MF prepolymer polymerizes and deposits onto these droplets. As the process continues, the droplets gradually develop into microcapsules with thickening MF shells. Further polymerization and crosslinking on the surface of the microcapsules result in the formation of a homogeneous shell, characterized by the uniformly distributed resin across the entire surface of the microcapsule. Therefore, achieving the desired shell thickness, density, and homogeneity requires careful balancing of the rates of MF resin polymerization, crosslinking, and deposition.

The choice of surfactant used for dispersing the selected PCM is also crucial [48,60,61,62]. To ensure the successful formation of a homogeneous and compact MF shell, the use of anionic emulsifiers is essential. The ionic attractions between the negatively charged head of the anionic surfactant and the positively charged MF resin result in a higher resin concentration at the surface, thereby accelerating the formation of the MF shell [47,60]. A higher MF resin concentration leads to faster polymerization. Additionally, the formation of the MF shell progresses more rapidly at lower pH values. However, excessively acidic conditions can increase the shell porosity and/or produce raspberry-like microcapsule shells [40]. On the other hand, higher pH values during microencapsulation result in a lower degree of crosslinking, producing mechanically unstable and flexible shells that are prone to deformation and cracking [52]. Therefore, establishing a balance between these factors is crucial for producing stable and efficient MF microcapsules.

In our previous research [63,64], we focused on preparing MF microcapsules at different temperatures and pH values. Microencapsulation conducted at lower pH values and higher temperatures resulted in thicker shells. It was demonstrated that lower pH values led to a higher degree of crosslinking of the MF resin. To enhance the thermal stability of the microcapsules, it was crucial to achieve a slow curing reaction and gradual deposition of the MF resin onto the microcapsule surface. We accomplished this by either gradually lowering the pH value in small steps or lowing it continuously throughout the microencapsulation process. The continuous, linear decrease in pH value resulted in microcapsules with thicker, harder MF shells and improved their thermal stability.

The main objective of this study, building on our previous research, was to further improve the thermal stability of MF microcapsules with a butyl stearate core by optimizing the microencapsulation and post-treatment processes. We hypothesized that the thermal stability of MF microcapsules could be improved by carefully controlling the thickness, porosity, and crosslinking of the MF shell. Specifically, we proposed that increasing the pH value during the early phase of microencapsulation would result in a thicker and more homogeneous MF shell. Recognizing that factors such as the temperature, stirring, concentration of reactants, and process duration also significantly influence shell properties, we kept these parameters constant to isolate and examine the specific impact of varied pH values on the shell characteristics. To test this, we used a commercially available etherified melamine–formaldehyde resin that exhibits rapid acid-catalyzed curing at relatively low temperatures [57,65] and high pH values. The use of a high starting pH value (6.0), which is higher than the starting values reported in other studies, was crucial for our approach, wherein different pH regimes were tested.

To our knowledge, the stepwise increase in pH value during microencapsulation, as applied in this study, has not been documented in the literature so far. To obtain comprehensive information on the effects of selected process parameters on microcapsule formation, samples were collected at different stages of the microencapsulation process. These samples were analyzed using differential scanning calorimetry (DSC), scanning electron microscopy (SEM), and thermogravimetric analysis (TGA).

## 2. Materials and Methods

### 2.1. Materials

For the microencapsulation process, a commercially available methanol etherified melamine–formaldehyde (MF) prepolymer (70 wt%, Melapret NF70/M; Melamin, Kočevje, Slovenia) with an F/M ratio of 2.5 was selected due to its exceptionally low free formaldehyde content. This choice was made to minimize the risk of variations in composition and/or structure that could affect the microencapsulation process. As the PCM, technical-grade butyl stearate (40–60%, with the rest being butyl palmitate; Sigma-Aldrich, Steinheim, Germany) with a melting enthalpy of 146 J/g was chosen. Sodium dodecyl sulfate (SDS, 98.5%; Sigma-Aldrich, Steinheim, Germany) was used as the surfactant. The pH value was regulated using formic acid (99.8%; Kemika, Zagreb, Croatia) and sodium hydroxide (99%; Merck, Darmstadt, Germany).

### 2.2. Preparation of Microcapsules

The preparation of the PCM suspension in water and the microencapsulation process were conducted in a 500 mL six-neck glass reactor. The reactor was equipped with a reflux condenser, an overhead stirrer with a Rushton turbine, a digital thermometer, a high-pressure liquid chromatography (HPLC) pump (K-120, Knauer, Berlin, Germany), an Ultra Turrax IKA T25 dispenser with an S 25 N–18 G dispersing tool, and a Mettler Toledo pH meter with an InLab Expert Pro pH sensor.

Butyl stearate (25 g) was dispersed in 400 g of a warm 0.625 wt% aqueous SDS solution using the Rushton turbine at 500 and the Ultra Turrax IKA T25 at 3400 rpm. During the first 30 min of stirring and dispersing, the suspension was heated to 70 °C. Afterward, 17.86 g of the MF prepolymer Melapret NF 70M, diluted with 57.14 g of water, was added. Stirring and dispersing continued for an additional 10 min, after which the Ultra Turrax was removed. The pH value of the suspension was then adjusted to 6.0 by adding a 1 wt% formic acid solution using the HPLC pump. Lowering the pH value to 6.0 triggered the polymerization of the MF resin, initiating the microencapsulation process.

During the microencapsulation process, the content of the reactor was stirred at 500 rpm and kept at a constant temperature of 70 °C. To investigate the effects of different pH value adjustment strategies, two microencapsulation processes, referred to as MC1 and MC2, were performed.

In the first stage of the MC1 series, which lasted 180 min, the pH value was allowed to rise spontaneously to approximately 6.5. Subsequently, the pH value was lowered to 5.0 by adding a 1 wt% formic acid solution at various flow rates (0.1, 0.2, or 0.4 mL/min). Once a pH value of 5.0 was reached, the process continued for an additional 60 min.

**Figure 1:**
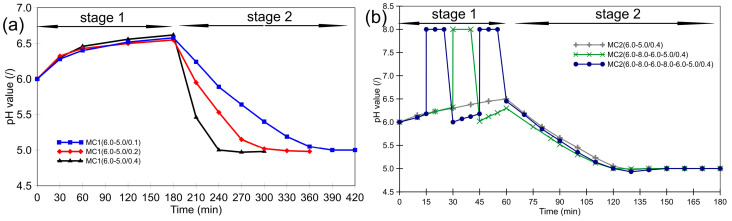
Changes in pH value in (**a**) the MC1 series and (**b**) the MC2 series of the microencapsulation experiments.

In the MC2 series, the initial stage was shortened to 60 min. The pH value was first raised to 8.0 and then lowered again to 6.0. Lowering the pH value to 5.0 was achieved by adding formic acid solution at a constant flow rate of 0.4 mL/min. After reaching a pH value of 5.0, the process continued for an additional 60 min without further pH control. A detailed scheme of the pH value adjustments during the MC1 and MC2 processes is shown in Figure 1. The microcapsule samples are referred to using the labels MC1(x–y/z) and MC2(x–y/z), where ‘x–y’ represents the pH value changes during the microencapsulation, and ‘z’ represents the flow rate of the 1% formic acid solution used to adjust the pH value to 5.0.

**Figure 2:**
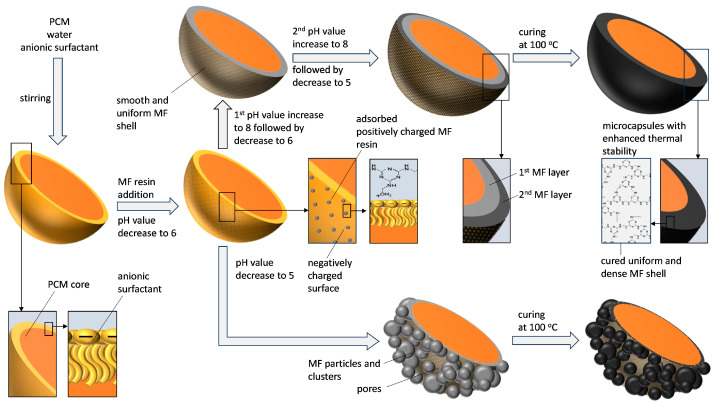
Schematic representation of the microencapsulation process for producing MF microcapsules with enhanced thermal stability for latent heat storage applications.

The final products were subjected to various post-curing treatments in a convection oven to increase their thermal stability [66]. The MC1 products were subjected to different post-curing temperatures: 100 °C, 125 °C, and 150 °C. The MC2 products were post-cured at 100 °C for 120 min. A schematic representation of the microencapsulation process is shown in Figure 2.

### 2.3. Characterization of the Microcapsules

The progress of the microencapsulation process was monitored by sampling and analyzing the reactor content throughout the process. Before the DSC analysis, TGA, and SEM analysis, these samples were filtered through Sartorius 388 paper filter disks and rinsed with warm deionized water. The resulting wet cake, containing MF microcapsules of butyl stearate and free of unencapsulated butyl stearate, was dried at room temperature until a constant weight was achieved.

#### 2.3.1. DSC Analysis

Differential scanning calorimetry (DSC) was used to determine the mass fractions of PCM and MF resin in the dried microcapsule samples. The amount of PCM was calculated from the melting enthalpies of bulk and encapsulated butyl stearate. The melting enthalpy of bulk butyl stearate was 146 J/g. The amount of MF resin was then calculated by subtracting the wt% of butyl stearate from 100 wt%.

Measurements were performed using a Mettler Toledo DSC1 instrument equipped with liquid nitrogen cooling and STAR software. Standard 40 μL aluminum pans were used. The analysis was conducted in an air atmosphere. The temperature program included an initial isothermal segment at 50 °C for 5 min, followed by a cooling segment from 50 °C to −50 °C at a rate of 5 °C/min. This was followed by a heating segment from −50 °C to 50 °C at the same rate. Each sample was analyzed three times and the average wt% of the core material was calculated from the melting enthalpies of the free and encapsulated core material in the third thermogram segment.

#### 2.3.2. SEM Analysis

The morphology of the microcapsules, including their size, shell homogeneity/smoothness, and thickness, along with other MF resin deposits, was analyzed using scanning electron microscopy with an FE-SEM ULTRA Plus instrument (Carl Zeiss, Oberkochen, Germany). The samples were partially cut with a razor blade to determine the thickness of the shell. They were mounted on carbon tape on aluminum pins, dried in a vacuum at 100 °C for 48 h, and then sputtered with gold. Imaging was performed at an accelerating voltage of 1 kV. The thickness of the shell was determined by taking at least four measurements for each sample to ensure sufficient accuracy and reliability. The average value was then calculated from these measurements.

#### 2.3.3. Thermogravimetric Analysis

Thermogravimetric analysis (TGA) of the isolated microcapsules was conducted to assess their thermal stability. The weight loss caused by the evaporation of butyl stearate and the degradation of MF resin was monitored. The thermal properties of the initial materials (butyl stearate and MF resin) were investigated in detail in our previous publication [64]. A Mettler Toledo TGA/DSC1 instrument with STAR software was used for the analysis. All experiments were carried out in a nitrogen atmosphere at a constant flow rate of 50 mL/min, with the temperature increasing from 50 °C to 500 °C at a rate of 10 °C/min. Standard 80 μL alumina (Al_2_O_3_) crucibles were used for the measurements.

## 3. Results and Discussion

### 3.1. Morphology of the Microcapsules during the Encapsulation Process and Post-Curing

**Figure 3:**
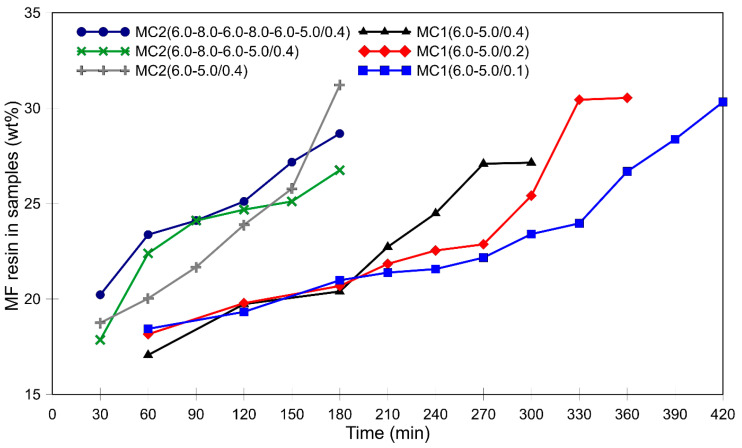
Weight percentage of MF resin in the samples during the microencapsulation process, determined by DSC analysis.

To monitor the deposition of the MF resin prepolymer and the shell formation during microencapsulation, filtered, rinsed, and dried microcapsules were analyzed using DSC. The wt% of PCM in the samples was calculated based on the measured melting enthalpy of the PCM core material, while the wt% of MF resin was determined by subtracting the wt% of PCM from 100%. Figure 3 shows the increase in the wt% of MF resin in the samples over time and highlights the differences between the two microencapsulation series. The observed variations in the increase indicate different rates of MF deposition. In both microencapsulation series, i.e., MC1 and MC2 (Figure 3), more than half of the final shell material was formed within the first 60 min of the process.

In the MC1 series, the pH value increased spontaneously from 6.0 to 6.5 during the first 180 min (Figure 1a). At these pH values, the formation of MF shell material was relatively slow, as shown in Figure 3. During the second and third hours, the amount of MF resin only increased by about 2–3 wt%. In the second stage, when the pH value was continuously lowered to 5.0, the rate of increase in the weight fraction of MF resin depended on the rate of pH value decrease. A slower decrease in pH value resulted in a higher weight fraction of MF material at the end of the second stage. The slower the pH value dropped, the longer the second stage lasted. After the pH value reached 5.0, the process continued for an additional hour, during which the maximum wt% of MF resin was reached. The increase in the wt% of MF resin in the samples is likely due to the thickening of the shells. Alternatively, it could also be due to the formation of pure MF particles (without the core PCM), which could deposit on the surface of the microcapsules and/or form MF resin agglomerates.

SEM analysis was used to assess the deposition of pure MF particles during microencapsulation and to evaluate the effects of different pH regimes on the microcapsules’ size and morphology. Most microcapsules had a diameter ranging from 2 to 10 μm, with the largest microcapsules reaching a diameter of approximately 20 μm. The size and distribution of the microcapsules were influenced by the method used to prepare the dispersion, as described in the Materials and Methods section. Since the dispersion preparation step was consistent across all experiments, no significant differences in particle size or distribution were anticipated when comparing microcapsules prepared under different pH regimes.

**Figure 4:**
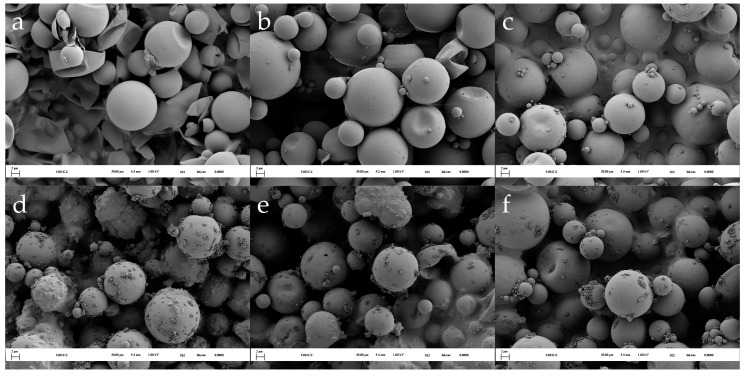
Morphology of isolated MC1 products: (**a**) MC1(6.0–5.0/0.1) at 30 min, (**b**) MC1(6.0–5.0/0.1) at 60 min, (**c**) MC1(6.0–5.0/0.1) at 180 min, (**d**) MC1(6.0–5.0/0.1) at 420 min, (**e**) MC1(6.0–5.0/0.2) at 360 min, and (**f**) MC1(6.0–5.0/0.4) at 300 min.

The results of the SEM analysis for the MC1 series are shown in Figure 4. After 30 min of the microencapsulation process (MC1(6.0–5.0/0.4) at 30 min, Figure 4a), severely damaged and deformed microcapsules were observed. It is likely that the shells of these microcapsules were not thick or mechanically stable enough to withstand the stress during the isolation process. In contrast, intact and undeformed capsules were isolated after 60 min of the microencapsulation process (MC1(6.0–5.0/0.4) at 60 min, Figure 4b).

Initiation of the microencapsulation at a pH value of 6.0 resulted in the formation of smooth-shelled microcapsules with minimal presence of small MF particles on the surface, even after 180 min (Figure 4c). The subsequent lowering of the pH value, controlled by the addition of formic acid, significantly influenced the polymerization of the MF resin prepolymer and its deposition (Figure 4d–f). The lowering of the pH value led to an uneven distribution of the MF resin, resulting in surface irregularities, such as larger clusters of resin-impregnated MF particles and aggregates of small spherical MF particles on the microcapsule surfaces (MC1(6.0–5.0/0.1) at 420 min, Figure 4d). Interestingly, increasing the flow rate of the acid decreased the occurrence of surface irregularities. For example, in MC1(6.0–5.0/0.4) (Figure 4f), the microcapsules exhibited a smoother surface with significantly fewer isolated or agglomerated MF particles.

A rapid decrease in the pH value effectively prevented significant and irreversible depositions of pure MF particles on the surface of the microcapsules that could not be separated during the isolation process. When the pH value was lowered slowly, the MF resin prepolymer and spherical particles with a lower degree of polymerization were deposited on the microcapsule shells, where the polymerization process continued. In contrast, when the pH value was lowered more rapidly, the polymerization progressed faster. This reduced the likelihood of MF particles with a higher degree of polymerization either undergoing crosslinking reactions with the microcapsule shell material or becoming trapped in newly deposited MF material on the microcapsule surface.

**Figure 5:**
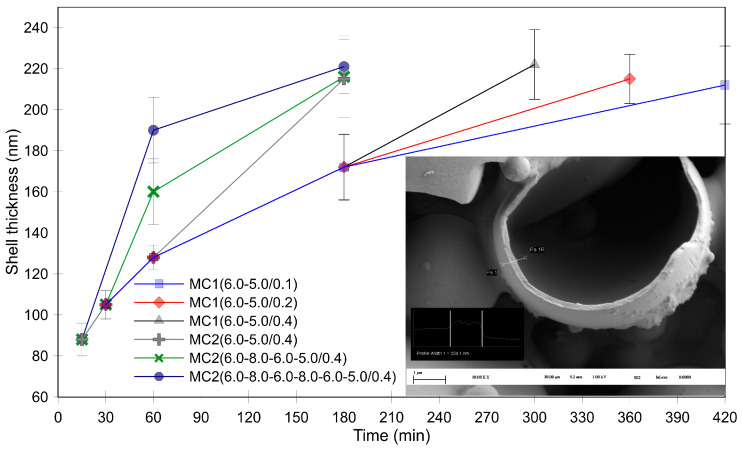
Microcapsule shell thicknesses for the MC1 and MC2 series.

In the next step, we estimated the shell thickness by examining intentionally damaged/broken isolated microcapsules using SEM. The results for the MC1 series (Figure 5) indicated that the shell thickness increased to approximately 170 nm within the first 180 min of the experiment. The final average shell thickness ranged from 210 to 220 nm and was somewhat influenced by the rate of the pH value decrease. A faster decrease in the pH value led to thicker shells. Notably, Figure 3 shows the opposite trend: a slower decrease in the pH value resulted in a higher wt% of MF resin in the isolated product. This observation can be explained by the fact that a rapid pH value decrease prevented a large amount of small, pure MF particles and MF agglomerates from attaching to the microcapsule surface (see Figure 4d–f).

The isolated MC1 microcapsules were subjected to various post-curing conditions at elevated temperatures. Elevated temperatures are necessary to complete the crosslinking of the MF resin, which involves the reaction of the methylol groups to form methylene ether and methylene bridges. The degree of crosslinking directly influences the mechanical properties of the MF resin. It is worth noting that the crosslinking process of the MF resin prepolymer used in this study has been investigated in previous research [59,63].

**Figure 6:**
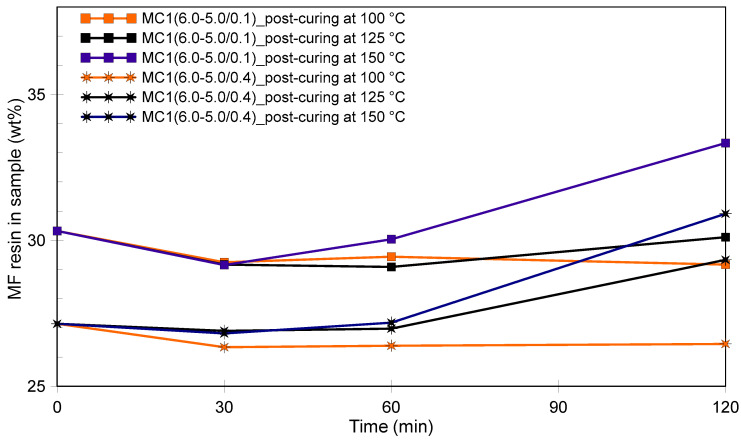
Weight percentage of the MF resin in the products MC1(6.0–5.0/0.1) and MC1(6.0–5.0/0.4) during post-curing under different conditions.

The microcapsules were exposed to temperatures of 100 °C, 125 °C, and 150 °C, with a maximum post-curing time of 2 h. After post-curing, they were analyzed again using DSC to determine their final PCM wt%. Figure 6 illustrates the changes in the MF resin wt% in the cured products MC1(6.0–5.0/0.1) and MC1(6.0–5.0/0.4). As expected, the wt% of MF resin in the samples decreased slightly due to the release of water, methanol, and formaldehyde—all volatile byproducts of the MF curing reaction. However, after prolonged post-curing, especially at higher temperatures, an increase in the MF resin wt% was observed. This indicates that PCM might have evaporated from mechanically damaged microcapsules or through permeable shells. Given that butyl stearate has a flash point of 160 °C [67,68], post-curing at 150 °C leads to higher pressure inside the microcapsules, potentially causing them to burst, especially if they are mechanically weaker. This explains why post-curing at 150 °C had a negative or at least no positive overall effect on the properties of the microcapsules.

The experimental design for the MC2 series (Figure 1b) was based on the following key observations from the MC1 series: (i) mechanically stable microcapsule shells formed after 60 min at pH value between 6.0 and 6.5, (ii) the addition of formic acid at 0.4 mL/min resulted in microcapsule surfaces with fewer irregularities and fewer adsorbed MF particles, and (iii) prolonged post-curing at elevated temperatures led to PCM losses. Recognizing the importance of the first 60 min, the focus was placed on enhancing the properties of the microcapsules during this period. The hypothesis was that increasing the pH value during the early phase of microencapsulation could result in a thicker, more homogeneous, and less porous MF shell.

Therefore, we adjusted the pH value during the first 60 min by first raising it from 6.0 to 8.0, keeping it at 8.0 for 10 min, and then lowering it back to 6.0. This stepwise approach aimed to control the deposition and polymerization rate of the MF resin on the microcapsule surface, which was coated with an anionic surfactant. We anticipated that increasing the pH value to 8.0 would reduce the polymerization rate and increase the MF resin concentration on the microcapsule surface. Consequently, the subsequent lowering of the pH value was expected to accelerate the condensation of the MF resin. The rate of condensation is influenced by both the pH value and the resin concentration: a higher concentration results in a faster reaction rate. Thus, this pH adjustment strategy can increase both the thickness of the shells and the degree of polymerization of the MF resin within them.

**Figure 7:**
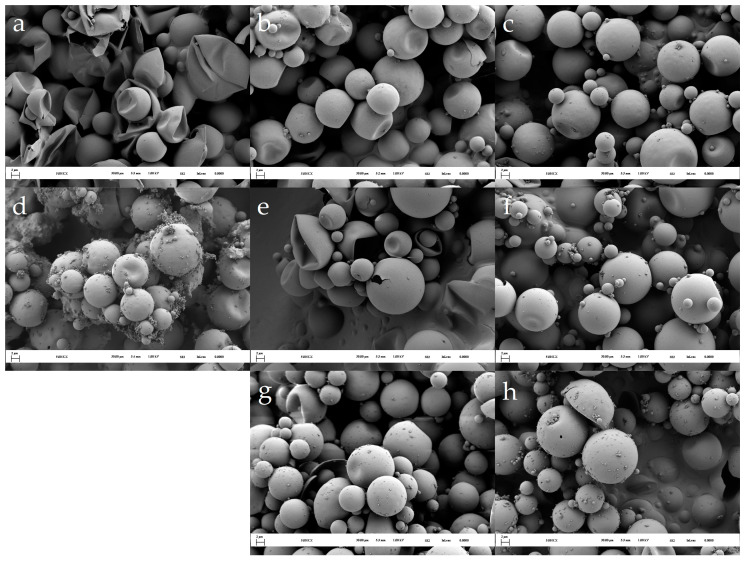
Morphology of the MC2 microcapsules: (**a**) MC2(6.0–5.0/0.4) at 15 min, (**b**) MC2(6.0–5.0/0.4) at 30 min, (**c**) MC2(6.0–5.0/0.4) at 60 min, (**d**) post-cured MC2(6.0–5.0/0.4), (**e**) MC2(6.0–8.0–6.0–5.0/0.4) at 60 min, (**f**) post-cured MC2(6.0–8.0–6.0–5.0/0.4), (**g**) MC2(6.0–8.0–6.0–8.0–6.0–5.0/0.4) at 60 min, and (**h**) post-cured MC2(6.0–8.0–6.0–8.0–6.0–5.0/0.4).

The positive effects of pH value modulation in the first 60 min are evident in Figure 3. Samples MC2(6.0–8.0–6.0–5.0/0.4) and MC2(6.0–8.0–6.0–8.0–6.0–5.0/0.4) showed a higher wt% of MF resin compared to MC2(6.0–5.0/0.4) and the MC1 samples. Increasing the pH value to 8.0 twice resulted in an even higher percentage of MF resin in the microcapsules. SEM analysis of these microcapsules revealed smooth surfaces with few small MF particles (Figure 7c,e,g), suggesting that the increased MF resin content was due to thicker shells. However, it is important to note that these samples also contained deformed microcapsules, likely due to the lower degree of polymerization of the MF resin at higher pH values.

The stepwise increase(s) in pH value significantly affected the shell thickness during the first 60 min (Figure 5). The shell thickness increased from 128 nm for MC2(6.0–5.0/0.4) to 160 nm with a single increase in pH value to 8.0 (MC2(6.0–8.0–6.0–5.0/0.4)). A second increase in the pH value further thickened the shell by 30 nm, resulting in a thickness of 190 nm for MC2(6.0–8.0–6.0–8.0–6.0–5.0/0.4). Thus, raising the pH value to 8.0 facilitated the formation of an additional MF layer with a lower degree of polymerization around the existing microcapsule shell.

In the second stage, the pH value was decreased to enhance the degree of polymerization and crosslinking of the resin. Interestingly, the shell thickness continued to increase during this stage, resulting in similar final shell thicknesses (215–220 nm, as shown in Figure 5) across different products, despite the variations in pH values in the first stage. However, while the final shell thicknesses were similar, it cannot be concluded that the thermomechanical properties of these microcapsules were also similar. The different pH values could have led to significantly different degrees of polymerization and crosslinking of the MF resin, potentially resulting in varying homogeneity and porosity of the shells in the final products. We hypothesize that a higher degree of polymerization of the MF resin deposited on the microcapsule surface would lead to increased porosity in the forming shell. Accordingly, notable differences in morphology were observed when comparing the post-cured products of the MC2 series (Figure 7).

The post-cured microcapsules of the MC2(6.0–8.0–6.0–5.0/0.4) (Figure 7f) and MC2(6.0–8.0–6.0–8.0–6.0–5.0/0.4) (Figure 7h) samples exhibited smooth surfaces with some small MF particles adhering to them. In contrast, the post-cured microcapsules of the MC2(6.0–5.0/0.4) sample (Figure 7d) had several MF particles and significantly larger, irregular clusters of MF resin on their surfaces.

### 3.2. Thermal Stability of Microcapsules and the Effect of Post-Curing

In thermogravimetric analyses (TGAs) of MF microcapsules with a butyl stearate core, weight loss occurs mainly due to the evaporation and degradation of the core material. Additionally, weight loss is observed due to processes such as polycondensation, crosslinking, and thermal degradation of the MF shell material [59,69,70]. Our previous research [64] provides a detailed description of the thermal degradation of MF microcapsules with a butyl stearate core.

**Figure 8:**
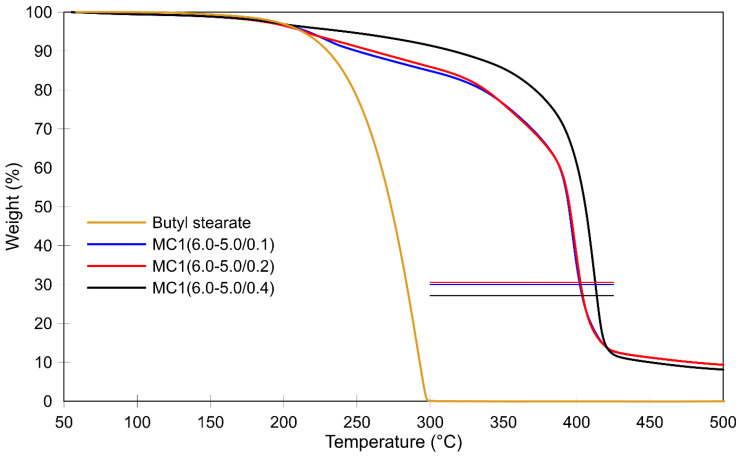
Thermal stability of MC1 samples before post-curing.

The MF resin prepolymer used here exhibits two significant weight loss stages. The first occurs at around 160 °C and is due to the evaporation of methanol, formaldehyde, and water during the curing reactions, while the second (at over 350 °C) is due to the degradation of the methylene bridges [59]. At temperatures above 400 °C, volatile decomposition products such as diazomethane, formaldehyde, CH_3_CN, HCN, and NH_3_ are released. The non-volatile residue contains melamine rings bridged with –NH– groups and various cyameluric structures [69,70]. In an MF resin with a higher degree of polymerization, these weight loss steps extend to higher temperatures. No TGA of isolated MF resin from the final product is reported in this study, as it was not possible to remove butyl stearate by Soxhlet extraction from the intentionally crushed microcapsule samples. Pure butyl stearate decomposes in a single step and evaporates at temperatures of 160 °C to 300 °C, leaving no residue, as shown in Figure 8. The shell of the microcapsule acts as a barrier, preventing the evaporation of the core material. Consequently, the reduced weight of the microcapsules caused by butyl stearate evaporation is influenced by the porosity and mechanical properties of the shell. The thermal degradation of the MF shell, which leads to shell breakage, begins above 350 °C.

Figure 8 shows the impact of the pH value decreasing from 6.0 to 5.0 on the thermal stability of the MC1 series. At 300 °C, when all of the non-encapsulated core material was completely vaporized, the total weight loss was only 15% or less. The horizontal lines represent the initial amount of shell material in a sample (around 30%). The results indicate that encapsulated butyl stearate is not completely removed from the microcapsules at temperatures below 400 °C. At 500 °C, the observed residue is approximately 10%, which likely corresponds to the remaining MF resin after thermal degradation. Based on our previous findings [64], the residue of pure MF resin at 500 °C is expected to be around 30–33%. Given that our samples contain 30% MF resin, this would account for about 9–10% of the total sample residue, as shown in Figure 8.

Among the MC1 series, the MC1(6.0–5.0/0.4) sample, characterized by the thickest shells, minimal surface irregularities (such as MF particles and unevenly deposited MF resin), and the lowest wt% of MF resin, exhibited the best thermal stability. This sample showed a weight loss of less than 10% at 300 °C, indicating a significantly improved thermal stability compared to our previous research [64], wherein a 17% weight loss was reported at the same temperature. In MC1(6.0–5.0/0.4), the thermal degradation of the methylene bridges, which led to shell fracture, occurred at higher temperatures. When comparing the TGA thermogram of MC1(6.0–5.0/0.4) with those of MC1(6.0–5.0/0.1) and MC1(6.0–5.0/0.2), a shift of about 10 °C towards higher temperatures was observed. However, the exact extent of this shift is difficult to determine accurately because the thermograms of MC1(6.0–5.0/0.1) and MC1(6.0–5.0/0.2) show significant weight loss due to incomplete crosslinking reactions. It is likely that MC1(6.0–5.0/0.4) had a higher degree of polymerization of its MF resin and a significantly higher proportion of methylene ether bridges, which were converted to methylene bridges before the TGA.

**Figure 9:**
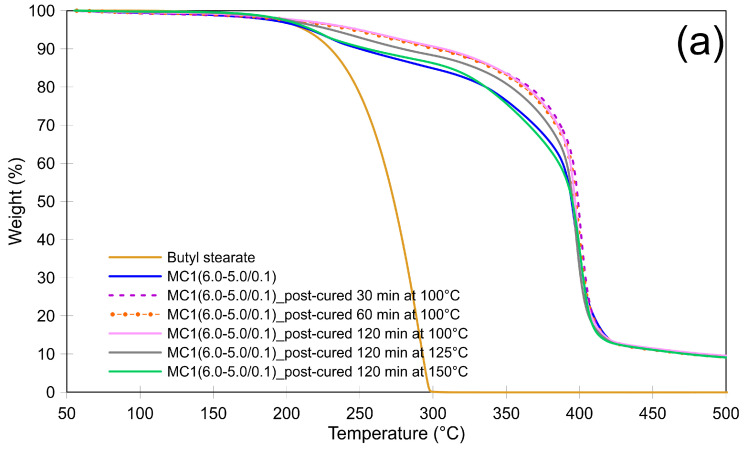

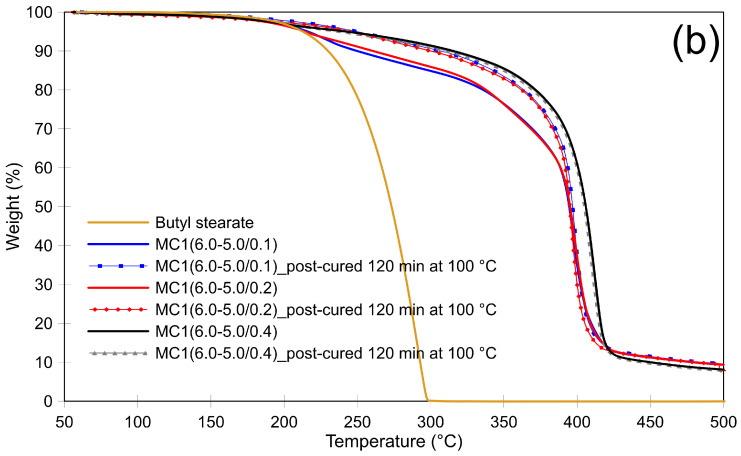
TGA curves for post-cured MC1 products: (**a**) Effects of different post-curing conditions on the thermal stability of MC1(6.0–5.0/0.1) microcapsules; (**b**) comparison of MC1 products with post-cured MC1 products at 100 °C for 120 min.

As mentioned earlier (Figure 6), exposing MC1 microcapsules to a post-curing temperature of 100 °C resulted in a slight decrease in the wt% of the MF shell. However, when the microcapsules were exposed to temperatures of 125 °C and 150 °C for more than 60 min, there was also a noticeable decrease in the wt% of the core material. These findings align with the TGA data presented in Figure 9a, which illustrate the effects of different post-curing conditions on thermal stability. Post-curing at 100 °C improved the thermal stability of the shells, with no noteworthy impact observed when extending the curing time from 30 to 120 min. Post-curing at 125 °C resulted in a modest increase in thermal stability, though it was not as significant as the improvement observed at 100 °C. Post-curing at 150 °C did not lead to an improvement. During post-curing at elevated temperatures, the following competing processes occur: (i) crosslinking reactions improve the thermomechanical stability of the MF resin; (ii) the transition of butyl stearate from the liquid to the gaseous phase can damage the shells of the microcapsules, particularly during prolonged curing; and (iii) the gaseous core material can migrate through porous shells. Therefore, the benefits of improved crosslinking must be carefully weighed against the potential risks of shell damage and core material loss.

Figure 9b shows the effects of a 120 min post-curing process at 100 °C on all MC1 products. Post-curing improved the thermal stability of samples MC1(6.0–5.0/0.1) and MC1(6.0–5.0/0.2) in the temperature ranges associated with crosslinking reactions. The weight losses observed in these samples were comparable to those in MC1(6.0–5.0/0.4). Interestingly, there was no noteworthy difference between the thermograms of MC1(6.0–5.0/0.4) before and after post-curing, suggesting that the microcapsule shells in this sample had already reached a high degree of crosslinking during the microencapsulation process. Furthermore, analysis of the TGA curves for the post-cured samples MC1(6.0–5.0/0.1) and MC1(6.0–5.0/0.2) revealed no shift in the onset of thermal degradation of the methylene bridges towards the temperature onset observed for MC1(6.0–5.0/0.4). This lack of a shift supports the hypothesis that thicker and more homogeneous shells significantly increase the thermal stability of these microcapsules.

**Figure 10:**
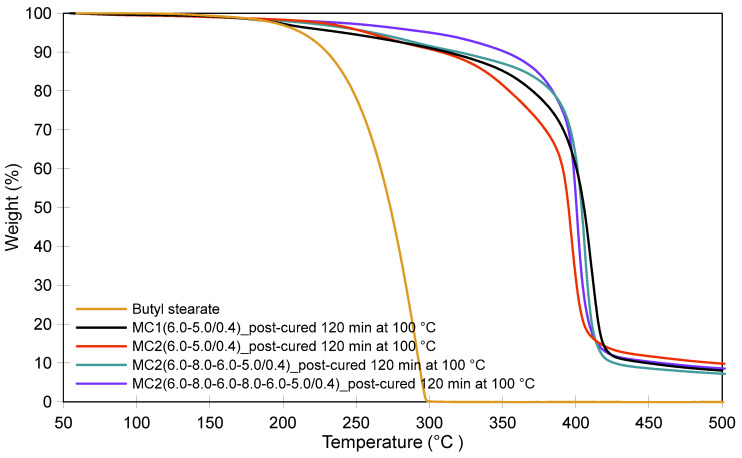
Thermal stability of the post-cured MC2 products compared to that of the post-cured product MC1(6.0–5.0/0.4).

Figure 10 compares the TGA thermograms of the post-cured MC2 samples with that of the post-cured sample MC1(6.0–5.0/0.4), which exhibited the best thermal properties among the MC1 samples. In the temperature range associated with crosslinking reactions and the conversion of methylene ether into methylene bridges, sample MC2(6.0–8.0–6.0–8.0–6.0–5.0/0.4) demonstrated superior thermal stability, which can be attributed to the higher crosslinking density of its MF shells. This higher crosslinking density was achieved by modulating the pH value within the first 60 min of the process. Each increase in the pH towards pH 8.0 resulted in a higher concentration of MF resin on the surface of the microcapsules, while each subsequent decrease in the pH towards pH 6.0 triggered condensation. Furthermore, as previously explained, when MF resin with a lower degree of polymerization is deposited on the microcapsule surface at higher pH values (8.0), the shell’s porosity is reduced. This lower porosity slows the diffusion of the core material through the shell at elevated temperatures, further contributing to the improved thermal stability.

In contrast, when analyzing the thermograms within the temperature range where the degradation of the methylene bridge occurs (Figure 10), the fastest loss of core material was observed in sample MC2(6.0–5.0/0.4). The higher weight loss in MC2(6.0–5.0/0.4) compared to MC1(6.0–5.0/0.4) can be attributed to the thicker shell of the MC1 sample at the end of the first stage. The thicker shell was formed during a much longer initial stage in which the pH spontaneously increased from 6.0 to 6.5. Additionally, the SEM image of the post-cured MC2(6.0–5.0/0.4) sample (Figure 7d) reveals an uneven deposition of MF resin on the surface of the microcapsules, characterized by large clumps of resin-impregnated MF particles forming agglomerates. This uneven deposition suggests that this MF resin had a higher degree of polymerization when it was deposited on the surface, leading to increased porosity in the shells. The higher porosity, in turn, contributed to the faster loss of the core material.

## 4. Conclusions

In summary, this study investigated different pH value regimes in the microencapsulation of butyl stearate via the in situ polymerization of melamine–formaldehyde resin and post-curing of the melamine–formaldehyde shells of the microcapsules. The goal was to develop microcapsules with optimal thermal stability. The findings confirmed our initial hypothesis that by controlling the thickness, homogeneity, and crosslinking of the MF shell, this goal could be achieved. Specifically, increasing the pH value during the early phase of microencapsulation resulted in thicker and more homogeneous MF shells.

The microencapsulation process was divided into two stages. The first stage, conducted at higher pH values, aimed to facilitate an efficient and uniform deposition of MF resin on the microcapsule surface. The optimal results were achieved by implementing two stepwise increases in the pH value until pH 8.0, followed by decreases to 6.0. The pH increases to 8.0 slowed the MF polycondensation rate, allowing MF resin with a lower degree of polymerization to migrate to the microcapsule surface. Subsequently, lowering the pH value to 6.0 accelerated polycondensation and crosslinking. In the second stage, the pH value was gradually reduced to 5.0, with the best results obtained using a formic acid flow rate of 0.4 mL/min, the fastest flow rate used in our study.

The optimized microencapsulation procedure resulted in accelerated shell formation, producing homogeneous, thick shells with significantly improved thermal stability. Post-curing at 100 °C was found to be optimal for enhancing thermal stability without causing damage to the microcapsule shell, which could occur during the transition of the core material from the liquid phase to the gaseous phase. However, prolonged curing at higher temperatures led to a loss of core material.

The post-cured microcapsules exhibited a total weight loss of less than 5% at 300 °C. Based on calculations of the microcapsules’ structure (core/shell ratio) and the weight loss of their pure components, it was determined that only 1% of the core material was released at this temperature.

In conclusion, this study presents a systematic and innovative approach to enhancing the thermal stability of PCM microcapsules. The findings offer valuable insights for applications requiring robust encapsulation techniques and superior thermal stability. Ensuring high thermo-mechanical resilience is especially crucial when incorporating PCM microcapsules into materials during their production processes, which often involve elevated temperatures and significant mechanical stresses. In the construction industry, the incorporation of MF-encapsulated PCMs into MF foams and other materials could be particularly beneficial.

## Data Availability

The data presented in this study can be made available on request from the corresponding author.
